# Role of protease-activated receptor-2 on cell death and DNA fragmentation in *Helicobacter pylori*-infected gastric epithelial cells

**DOI:** 10.1186/1479-5876-8-85

**Published:** 2010-09-16

**Authors:** Joo Weon Lim, Hyeyoung Kim

**Affiliations:** 1 Department of Food and Nutrition, Brain Korea 21 Project, College of Human Ecology, Yonsei University, Seoul 120-749, Korea

## Abstract

**Background:**

*Helicobacter pylori *(*H. pylori*) infection is associated with chronic gastritis, peptic ulceration and gastric carcinoma. Protease-activated receptor-2 (PAR-2), which is activated by trypsin, induced the activation of mitogen-activated protein kinases (MAPK), cell proliferation and apoptosis in several cells. Previously, we found that *H. pylori *induces the expression of PAR-2, which mediates the expression of adhesion molecules integrins in gastric epithelial cells. In the present study, the role of PAR-2 on *H. pylori*-induced cell death was investigated by determining cell viability, DNA fragmentation, and the activation of MAPK in gastric epithelial AGS cells.

**Methods:**

AGS cells were cultured in the presence of *H. pylori *transfected with PAR-2 antisense (AS) oligonucleotide (ODN) or treated with a soybean trypsin inhibitor (SBTI). Viable cells and DNA fragmentation were determined by trypan blue exclusion assay and the amount of oligonucleosome-bound DNA, respectively. The activation of MAPK such as extracellular signal-regulated kinases (ERK), p38, and c-Jun N-terminal kinases (JNK), was assessed by Western blotting for phospho-specific forms of MAPK.

**Results:**

*H. pylori*-induced cell death and DNA fragmentation augmented in the cells transfected with PAR-2 AS ODN or treated with SBTI. The activation of MAPK, induced by *H. pylori*, were suppressed by transfection with PAR-2 AS ODN or treatment with SBTI.

**Conclusion:**

PAR-2, whose expression is induced by *H. pylori*, may prevent cell death and DNA fragmentation with the activation of MAPK in gastric epithelial cells.

## Background

*Helicobacter pylori *(*H. pylori*) has been shown to be an important pathogen of gastroduodenal inflammation and gastric carcinogenesis [1,2]. *H. pylori *infection increases epithelial apoptosis in gastric mucosa, which may play an important role in gastric carcinogenesis [3]. *H. pylori*-induced apoptosis may stimulate compensatory hyperproliferation which results in potential preneoplastic changes in chronic *H. pylori *infection [4-6]. *H. pylori *-induced apoptosis has been shown in gastric epithelial cells [7,8] as well as infected gastric tissues [6,9,10]. However, the apoptotic mechanism induced by *H. pylori *infection has not been fully elucidated.

*H. pylori *activates three main groups of mitogen-activated protein kinases (MAPKs), i.e., the extracellular signal-regulated kinases 1 and 2 (ERK1/2), p38 MAPKs, and c-Jun N-terminal kinases [11,12]. Recently, it was shown that inhibition of the ERK1/2 pathway augmented *H. pylori*-induced apoptosis in gastric epithelial cells [13], demonstrating the possible involvement of MAPK in gastric apoptosis.

Proteinase-activated receptors (PARs), a family of G protein-coupled seven-*trans*-membrane domain receptors, mediate a variety of intracellular signaling and subsequent cellular events caused by specific extracellular proteinases [14,15]. The family of PARs currently includes four members: PAR-1, PAR-2, PAR-3 and PAR4. The coagulant protease thrombin is the physiological activator of PAR-1, PAR-3, and PAR-4. PAR-2 is activated by multiple trypsin-like serine proteases including trypsin, tryptase and coagulation protease upstream of thrombin. Activation of PAR-2 triggers the activation of multiple signaling pathways, including MAPK cascades in distinct cell types [16,17]. PAR-2 is involved in cell proliferation and apoptosis in several cell types [18,19]. Recent data suggest that activation of PAR-2 rescued cells from apoptosis via activation of MAPKs [20]. We previously demonstrated that *H. pylori *induces the activation and expression of PAR-2 in gastric epithelial cells [21,22]. These results demonstrate the possible relations of the expression of PAR-2, the activation of MAPK, and apoptosis in *H. pylori*-infected gastric epithelial cells. The present study aims to investigate whether *H. pylori*-induced apoptotic cell death is related to the expression of PAR-2 and the activation of MAPK in gastric epithelial cells.

## Methods

### Bacterial strain

An *H. pylori *strain used in the present study is HP99 isolated form Korean patients and identified as cagA+, vacA+ strain [12]. HP99 is kindly provided from Dr. H.C. Jung (Seoul National University College of Medicine, Seoul, Korea). These bacteria were inoculated onto chocolate agar plates (Becton Dickinson Microbiology Systems, Cockeysville, MD, USA) at 37°C under microaerophilic conditions using an anaerobic chamber (BBL Campy Pouchs System, Becton Dickinson Microbiology Systems).

### Cell culture and *H. pylori *stimulation

A human gastric epithelial cell line AGS (gastric adenocarcinoma, ATCC CRL 1739) was obtained from the American Type Culture Collection (Rockville, MD, USA). The cells were grown in complete medium, consisting of RPMI 1640 medium supplemented with 10% fetal bovine serum, 2 mM glutamine, 100 U/ml penicillin, and 100 μg/ml streptomycin (Sigma, St. Louis, MO, USA). AGS cells were seeded and cultured to reach 80% confluency. Prior to the stimulation, each dish was washed twice with fresh cell culture medium containing no antibiotics. *H. pylori *was harvested, washed with phosphate buffered saline (PBS), and then resuspended into antibiotic-free cell culture medium. *H. pylori *was added to AGS cells at bacterium/cell ratio of 150:1 or 300:1, and cultured for the indicated time periods.

### Experimental protocol

To investigate the relations of apoptotic cell death, the expression of PAR-2, and the activation of MAPK in *H. pylori*-infected gastric epithelial cells, cell viability and DNA fragmentation were determined in the cells transfected with PAR-2 AS ODN or S ODN or treated with soybean trypsin inhibitor (SBTI; 0.2 μM, 0.5 μM) and cultured in the presence of *H. pylori *at bacterium/cell ratio of 150:1 (cell viability) or 300:1 (cell viability, DNA fragmentation) for 24 hours. For the activation of MAPK, AGS cells were transfected with PAR-2 AS ODN or S ODN or treated with soybean trypsin inhibitor (SBTI; 0.2 μM, 0.5 μM) and cultured in the presence of *H. pylori *at bacterium/cell ratio of 300:1 for 30 minutes. To determine the transfection efficiency of PAR-2 AS ODN or S ODN, the level of PAR-2 was determined in the transfected cells cultured in the presence of *H. pylori *at bacterium/cell ratio of 300:1 for 2 hours.

### Determination of cell viability and DNA fragmentation

AGS cells were cultured in the presence of *H. pylori *at a bacterium/cell ratio of 150:1 or 300:1 for 24 hours. Viable cells were determined by trypan blue exclusion test (0.2% trypan blue). DNA fragmentation was determined by the amount of oligonucleosome-bound DNA in the cell lysates using a sandwich ELISA (Cell Death Detection ELISAplus kit; Boehringer-Mannheim). The relative increase in oligonucleosome-bound DNA was determined at 405 nm and expressed as an enrichment factor.

### Treatment with ODNs using cationic liposome

Single-stranded oligonuceltides (ODNs) were produced commercially (GIBCO BRL, Rockville, MD, USA). ODNs were phosphorothioate-modified to reduce intracellular nuclease digestion. Antisense (AS) and sense (S) ODNs targeted the ATG start codon of the human PAR-2 mRNA [GenBank: AY336105.1]. The sequence of the PAR-2 AS ODN was 5'TCCGCATCCTCCTGGAA3'. The sequence of PAR-2 S ODN was 5'TTCCAGGAGGATGCGGA3'. AGS cells were transfected with ODNs using a cationic liposome, a commercially available transfection-reagent DOTAP (N-[1-(2,3-dioleoyloxy) propyl]-N, N, Ntrimethyl ammonium methylsulfate) (Boehringer-Mannheim, Pentzberg, Germany) to improve stability and intracellular delivery of ODNs. When DOTAP was employed, the appropriate amount of ODNs were incubated with DOTAP (15 μl/ml) to achieve the respective final concentration of the ODNs to 0.5 μM at 37°C for 15 min. The cells were treated with the mixture and then incubated for 24 hours. After medium was changed with antibiotic-free medium, the transfected cells were cultured in the presence of *H. pylori*.

### Western blot analysis

One hundred μg of whole cell extracts was loaded per lane, separated by SDS-polyacrylamide gel electrophoresis (PAGE) under reducing conditions, and transferred onto Hybond-PVDF membranes (Amersham Inc., Arlington Heights, IL, USA) by electroblotting. The transfer of protein and equality of loading in all lanes was verified using reversible staining with Ponceau S. Membranes were blocked using 5% nonfat dry milk. PAR-2, ERK1/2, p38, and JNK1/2 were detected by incubation of blots with specific polyclonal antibodies, followed by sheep anti-mouse secondary antibody conjugated to horseradish peroxidase. The proteins were determined by enhanced chemiluminescence (Amersham) using exposure to BioMax MR film (Kodak).

### Statistical analysis

Results are expressed as means ± standard error of four separate experiments. Analysis of variance (ANOVA) followed by Newman-Keul's test was used for statistical analysis. *P* < 0.05 was considered statistically significant

## Results

### Inhibition of PAR-2 expression augments *H. pylori*-induced cell death and DNA fragmentation in gastric epithelial cells

**Figure 1:**
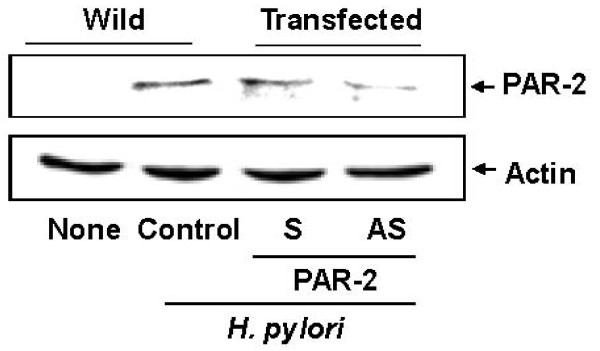
**Transfection of AS ODN for PAR-2 inhibits the expressiongn of PAR-2 in *H. pylori*-infected AGS cells**. AGS cells were transfected with PAR-2 AS ODN or S ODN for 24 hours and cultured in the absence or the presence of *H. pylori *at a bacterium/cells ratio of 300:1 for 2 hours. The levels of PAR-2 were determined by Western blot analysis using specific antibody for PAR-2. Actin served as a loading control. Wild, the cells without transfection; Transfected, the cells transfected with S ODN (S) or AS ODN (AS). None, the cells without transfection and cultured in the absence of *H. pylori; H. pylori *control, the cells without transfection and cultured in the presence of *H. pylori*.

**Figure 2:**
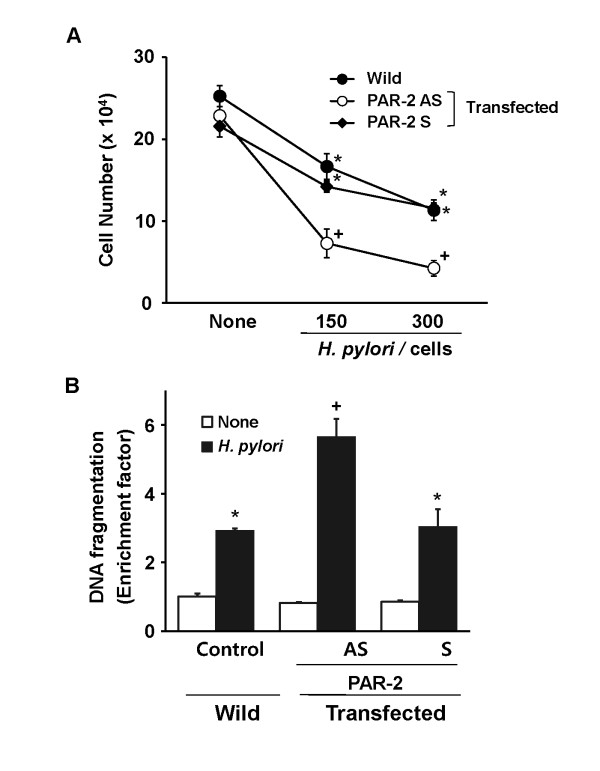
**Inhibition of PAR-2 expression augments *H. pylori*-induced cell death and DNA fragmentation in AGS cells**. (A) AGS cells were transfected with PAR-2 AS ODN or S ODN for 24 hours and cultured in the absence or the presence of *H. pylori *at a bacterium/cells ratio of 150:1 or 300:1 for 24 hours. Viable cell numbers were determined by trypan blue exclusion test. The results represent mean ± SE of four different experiments. *P < 0.05 compared to the corresponding none (the cells cultured in the absence of *H. pylori *). ^+^P < 0.05 compared to the corresponding wild or PAR-2 S (wild cells or the cells transfected with S ODN and cultured in the presence of *H. pylori *at a bacterium/cells ratio of 150:1 or 300:1). (B) AGS cells were transfected with PAR-2 AS ODN or S ODN for 24 hours and cultured in the absence or the presence of *H. pylori *at a bacterium/cells ratio of 300:1 for 24 hours. DNA fragmentation was determined by the amount of oligonucleosome-bound DNA in the cell lysates. The relative increase in oligonucleosome-bound DNA was determined at 405 nm and expressed as an enrichment factor. The results represent mean ± SE of four different experiments. *P < 0.05 compared to none control (the cells without transfection and cultured in the absence of *H. pylori*). ^+^P < 0.05 compared to wild *H. pylori *control (the cells without transfection and cultured in the presence of *H. pylori*). Wild, the cells without transfection; Transfected, the cells transfected with S ODN (S) or AS ODN (AS).

To investigate the relations of PAR-2 expression, cell death, and DNA fragmentation, cells were transfected with AS ODN for PAR-2 and cultured in the presence of *H. pylori*. As shown in figure 1, *H. pylori *induced the expression of PAR-2, which was inhibited in the cells transfected with PAR-2 AS ODN. Viable cell numbers were decreased by *H. pylori *with the number of bacterium infected to the cells (Figure 2A). Cell death of *H. pylori*-infected cells was augmented by transfection with PAR-2 AS ODN, compared with that of the cells transfected with S ODN or wild cells (non-transfected cells). Similarly, *H. pylori*-induced DNA fragmentations increased by transfection with PAR-2 AS ODN, compared with corresponding S ODN or wild cells (Figure 2B). These results suggest that *H. pylori- *induced expression of PAR-2 may have a protective role against apoptosis of gastric epithelial cells, determined by cell death and DNA fragmentation.

### Inhibition of PAR-2 expression suppresses *H. pylori*-induced activation of MAPK in gastric epithelial cells

**Figure 3:**
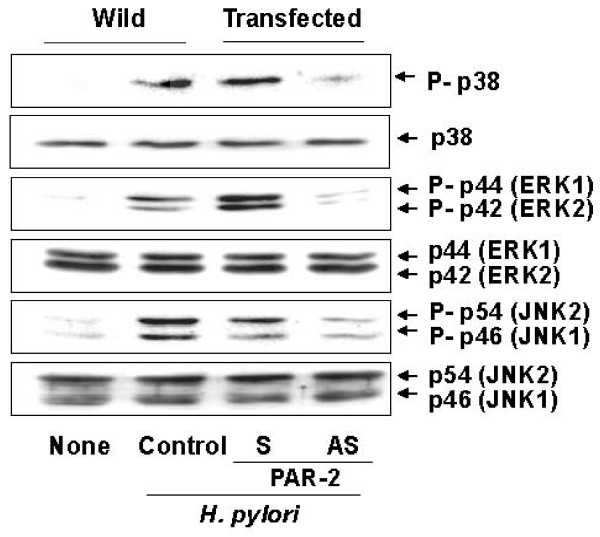
**Inhibition of PAR-2 expression suppresses *H. pylori*-induced activation of MAPK in gastric epithelial cells**. AGS cells were transfected with PAR-2 AS ODN or S ODN for 24 hours and cultured in the absence or the presence of *H. pylori *at a bacterium/cells ratio of 300:1 for 30 minutes. The levels of phospho-specific and total forms of MAPK (p38, ERK1/2, JNK1/2) were determined by Western blotting using specific antibodies for the indicated proteins. Wild, the cells without transfection; Transfected, the cells transfected with S ODN (S) or AS ODN (AS). None, the cells without treatment and cultured in the absence of *H. pylori; H. pylori *control, the cells without treatment and cultured in the presence of *H. pylori*.

To determine the role of PAR-2 on the activation of MAPK, the activation of three major MAPK involved in cell proliferation and apoptosis were assessed by Western blotting of phospho-specific and total forms of MAPK (p38, ERK1/2, JNK1/2). As shown figure 3, levels of phospho-specific forms of p38, ERK1/2, and JNK1/2 increased by *H. pylori *in AGS cells. *H. pylori *did not affect total forms of p38, ERK1/2, and JNK1/2 in AGS cells. *H. pylori*-induced activation of MAPK was inhibited in the cells transfected with PAR-2 AS ODN, but that was not affected in the cells transfected with S ODN. These results demonstrate that both *H. pylori*-induced expression of PAR-2 and activation of MAPK may be related to cell viability of gastric epithelial cells after *H. pylori *infection.

### Soybean trypsin inhibitor (SBTI) augments *H. pylori*-induced cell death and DNA fragmentation in gastric epithelial cells concentration-dependently

**Figure 4:**
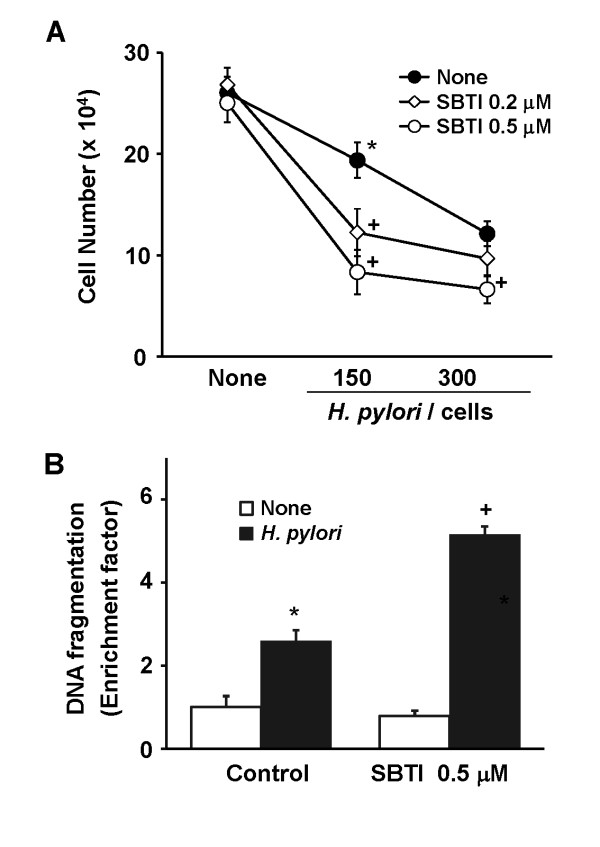
**Soybean trypsin inhibitor (SBTI) augments *H. pylori*-induced cell death and DNA fragmentation in gastric epithelial cells concentration-dependently**. (A) AGS cells were treated with SBTI (0.2 μM, 0.5 μM) and cultured in the absence or the presence of *H. pylori *at a bacterium/cells ratio of 150: or 300:1 for 24 hours. Viable cell numbers were determined by trypan blue exclusion test. The results represent mean ± SE of four different experiments. *P < 0.05 compared to none (the cells without treatment and cultured in the absence of *H. pylori*). ^+^P < 0.05 compared to the corresponding none (the cells without treatment and cultured in the presence of *H. pylori *at a bacterium/cells ratio of 150: or 300:1). (B) AGS cells were treated with SBTI (0.5 μM) and cultured in the absence or the presence of *H. pylori *at a bacterium/cells ratio of 300:1 for 24 hours. DNA fragmentation was determined by the amount of oligonucleosome-bound DNA in the cell lysates. The relative increase in oligonucleosome-bound DNA was determined at 405 nm and expressed as an enrichment factor. The results represent mean ± SE of four different experiments. *P < 0.05 compared to none control (the cells without treatment and cultured in the absence of *H. pylori*). ^+^P < 0.05 compared to *H. pylori *control (the cells without treatment and cultured in the presence of *H. pylori*).

Since PAR-2 is activated by trypsin [14,15], SBTI was treated to the cells and cultured in the presence of *H. pylori *to suppress the activity of PAR-2. Viable cell numbers were decreased by *H. pylori *with the number of bacterium infected to the cells (Figure 4A). Cell death of *H. pylori*-infected cells was augmented by treatment of SBTI concentration-dependently. *H. pylori*-induced DNA fragmentations increased by treatment of 0.5 μM of SBTI (Figure 4B). These results suggest that inhibition of trypsin activity, which induces the suppression of PAR-2 activity, augments *H. pylori- *induced cell death and DNA fragmentation.

### Soybean trypsin inhibitor (SBTI) suppresses *H. pylori*-induced activation of MAPK in gastric epithelial cells

**Figure 5:**
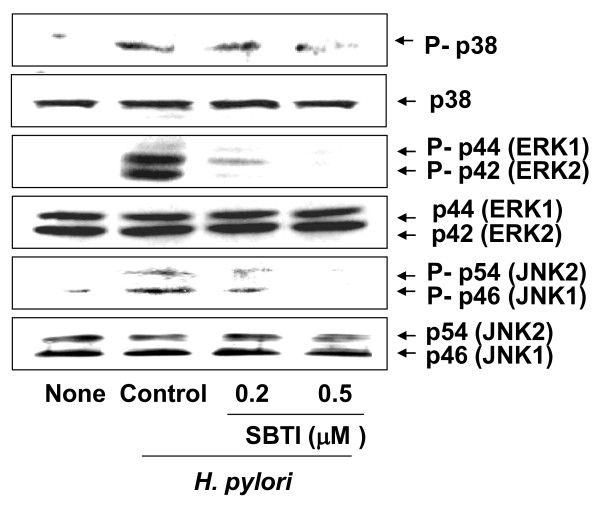
**Soybean trypsin inhibitor (SBTI) suppresses *H. pylori*-induced activation of MAPK in gastric epithelial cells**. AGS cells were treated with SBTI and cultured in the absence or the presence of *H. pylori *at a bacterium/cells ratio of 300:1 for 30 minutes. The levels of phospho-specific and total forms of MAPK (p38, ERK1/2, JNK1/2) were determined by Western blotting using specific antibodies for the indicated proteins. None, the cells without treatment and cultured in the absence of *H. pylori; H. pylori *control, the cells without treatment and cultured in the presence of *H. pylori*.

*H. pylori *-induced increases in phospho-specific forms of p38, ERK1/2, and JNK1/2 were inhibited by treatment by treatment of SBTI in AGS cells concentration-dependently (figure 5). As shown figure 3, *H. pylori *did not affect total forms of p38, ERK1/2, and JNK1/2 in AGS cells, which was not affected by treatment of SBTI (figure 5). These results suggest that PAR2, which may be activated by trypsin, may be involved in the activation of MAPK in *H. pylori*-infected gastric epithelial cells.

## Discussion

In the present study, we found that *H. pylori*-induced cell death and DNA fragmentation were augmented by inhibition of PAR-2 expression using PAR-2 AS ODN in AGS cells. Additionally, inhibition of PAR-2 activity using trypsin inhibitor increased cell death and DNA fragmentation in *H. pylori*-infected AGS cells. These results demonstrate that the expression and the activation of PAR-2 induced by *H. pylori *may prevent apoptotic cell death in gastric epithelial cells.

Previously we demonstrated that *H. pylori *induced the expression and activation of PAR-2, which mediates the expression of COX-2 and integrins in gastric epithelial cells [21,22]. Trypsin activated PAR-2, which mediated the proliferation of various cells including pancreatic and gastric cancer cells, and smooth muscle cells [23-25]. Additionally, activation of PAR-2 by agonist or activating peptide protects astrocytes and neutrophils against apoptotic cell death [26,27]. These studies support the present results showing that *H. pylori*-induced expression of PAR-2 may protect gastric epithelial cells from cell death and DNA fragmentation.

Furthermore, we here found that activation of MAPK was mediated by PAR-2 in *H. pylori*-infected gastric epithelial cells. This result is consistent with the previous studies showing that the activation of PAR-2 is related to the activation of MAPK in mouse tracheal and bronchial smooth muscle [16,17]. The MAPK signaling pathways play essential roles in cell proliferation and apoptosis [18,19]. MAPK activated by *H. pylori *infection is involved in apoptosis of gastric epithelial cells [28-30]. Ding et al. [28] demonstrated that inhibition on the activation of ERK1/2, JNK1/2 or p38 by treatment of the chemical inhibitor increased *H. pylori*-induced apoptosis in gastric epithelial cells. These studies suggest that PAR-2 activation induced by *H. pylori *may protect gastric epithelial cells from apoptosis by the activation of MAPK. In contrast, inhibition of p38 by a specific inhibitor SB203580 decreased apoptosis while ERK1/2 inhibition by a specific inhibitor PD98059 resulted in an increase of apoptosis in *H. pylori*-infected gastric epithelial cells [29]. Further studies should be performed to investigate the role of PAR-2 on the activation of specific MAPK and its relation to the expression of apoptotic genes in *H. pylori*-infected gastric epithelial cells. Recently it was reported that other PARs such as PAR-1 and PAR-4 protect cell apoptosis through ERK or JNK signaling pathway [30-34]. Therefore, the expression and activation of PAR-2 induced by *H. pylori *may rescue gastric epithelial cells from apoptosis via MAPK signaling.

Other possible protective proteins other than PARs, antiapoptotic proteins such as inhibitors-of-apoptosis-proteins (IAPs) were induced by NF-κB activation and protected the cells from apoptosis induced by the wild-type *H. pylori *containing virulence factor cytotoxin-associated gene (*cagA*) [35]. Molecular chaperone heat shock protein 70 protected gastric injury against monochloramine which is generated by neutrophil-derived hypochlorous acid and *H pylori *urease-induced ammonia [36]. *H. pylori *infection upregulated gastric mucosal peroxiredoxin (Prx) I expression, and further, that Prx I played an important role in gastric mucosal protection against oxidative injury induced by *H. pylori *infection [37]. Recently, it was found that the disturbances in gastric mucosal NO generation system caused by *H. pylori *resulted from the inducible nitric oxide synthase (iNOS). They demonstrated that peptide hormone ghrelin protected gastric mucosa from *H. pylori*-induced proapoptotic events by a decrease in S-nitrosylation of constitutive nitric oxide synthase (cNOS) [38].

Regarding virulence factors of *H. pylori*, *cagA *and vacuolating cytotoxin A (*vacA*) are reported to contribute to gastric cancer incidence [39,40]. Since *H. pylori *in Korean isolates, HP99 used in the present study, contains both *cagA *and *vacA *[41], the present result may explain the possible mechanism of *H. pylori*-induced gastric carcinogenesis. Further study should be performed to investigate the mechanism of gastric carcinogenesis involving PAR-2-associated prevention against apoptosis of gastric epithelial cells infected with *H. pylori *containing different isotypes of virulence factors.

## Conclusion

PAR-2, whose expression is induced by *H. pylori*, may prevent cell death and DNA fragmentation with the activation of MAPK in gastric epithelial cells. These results demonstrate a novel mechanism of protection against apoptotic cell death by PAR-2 in *H. pylori*-infected gastric epithelial cells.

## Abbreviations

AS: antisense; MAPK: mitogen-activated protein kinase; ODN: oligonucleotide; PAR: protease-activated receptor; S: sense; SBTI: soybean trypsin inhibitor

## Competing interests

The authors report no conflicts of interest. The authors alone are responsible for the content and writing of the paper.

## Authors' contributions

All authors read and approved the final manuscript.
